# Neuropathogenesis caused by *Trypanosoma brucei*, still an enigma to be unveiled

**DOI:** 10.15698/mic2021.04.745

**Published:** 2021-04-01

**Authors:** Katherine Figarella

**Affiliations:** 1 Department of Neurophysiology, Institute of Physiology, University of Tübingen, Germany.

**Keywords:** Trypanosoma, anti-trypanosomatid drugs, meningoencephalitis, neuropathogenesis, two-photon microscopy

## Abstract

*Trypanosoma brucei* is one of the protozoa parasites that can enter the brain and cause injury associated with toxic effects of parasite-derived molecules or with immune responses against infection. Other protozoa parasites with brain tropism include *Toxoplasma, Plasmodium, Amoeba*, and, eventually, other *Trypanosomatids* such as *T. cruzi* and *Leishmania*. Together, these parasites affect billions of people worldwide and are responsible for more than 500.000 deaths annually. Factors determining brain tropism, mechanisms of invasion as well as processes ongoing inside the brain are not well understood. But, they depend on the parasite involved. The pathogenesis caused by *T. brucei* initiates locally in the area of parasite inoculation, soon trypanosomes rich the blood, and the disease enters in the so-called early stage. The pathomechanisms in this phase have been described, even molecules used to combat the disease are effective during this period. Later, the disease evolves towards a late-stage, characterized by the presence of parasites in the central nervous system (CNS), the so-called meningo-encephalitic stage. This phase of the disease has not been sufficiently examined and remains a matter of investigation. Here, I stress the importance of delve into the study of the neuropathogenesis caused by *T. brucei*, which will enable the identification of pathways that may be targeted to overcome parasites that reached the CNS. Finally, I highlight the impact that the application of tools developed in the last years in the field of neuroscience will have on the study of neglected tropical diseases.

More than a century has passed since the discovery of the hemoflagellate *Trypanosoma brucei* (1895) and its identification as the causal agent of Human African Trypanosomiasis (HAT; 1902). Promptly could be established that the disease arises, depending on the parasite subspecies involved, in an acute form in the case of *Trypanosoma brucei rhodesiense* or a chronic form in the case of *Trypanosoma brucei gambiense*. Currently, the last accounts for 98% of reported cases. This great inequity in prevalence is largely due to the short survival time of infected individuals with *T. b. rhodesiense* (some months). It unequivocally reduces the probability for transmission but also may drop the chance for those cases to get registered. In the course of HAT, there are two well-differentiated stages. The early (haemolymphatic) stage is characterized by the presence of parasites in lymph and blood. Unfortunately, during this period, patients tend to show unspecific symptoms like headache, intermittent fever, malaise, arthralgia, weight loss, and fatigue, which complicates the early diagnosis and therefore the treatment, because most drugs available against the disease are effective only in this phase. The late meningo-encephalitic stage is defined after a lumbar puncture by the presence of parasites and/or elevated levels of white blood cells (≥ 5 WBC/mm^3^) in the cerebrospinal fluid (CSF) [1]. Clinical manifestations and symptoms of the second disease stage include severe sleep disturbances, neurological and psychiatric disorders, coma, and death if is left untreated.

The World Health Organization (WHO) started in the early 2000's an initiative towards eliminating HAT as a public health problem. Indeed, the number of cases of the disease has dropped in the last years. Nevertheless, there is still a threat of re-emergence as the disease has always appeared periodically with devastating outbreaks at the turn of the 20^th^ century, in the 1940ties and the last in the 1980ties. Also, external factors like socio-economic conditions or political instability may affect health services in endemic countries and therefore, control of the disease may decline. Molecules used to treat HAT were developed in the first half of the past century. Pentamidine (1937) and suramin (1916) are used exclusively to treat the first stage because they poorly cross the blood-brain barrier (BBB). Melarsoprol (1949) or a combination of eflornithine (1970) and nifurtimox (1965) (NECT, introduced in 2009) are administrated to patients in the meningo-encephalitic stage [2]. NECT is been currently used in areas affected by *T. b. gambiense*. Nonetheless, this chemotherapy is suboptimal not only because its administration is painful, long-lasting, and highly complex, requiring sophisticated logistics and nursing care, but also because of its elevated toxicity. In 2019, the WHO introduced fexinidazole as a new treatment for both stages of gambiense HAT. Undoubtedly it has represented an improvement in the disease's therapeutic due to its oral administration. However, it even needs the supervision of trained medical staff. Currently, another molecule of the oxaborole group (acoziborole) is being evaluated against both HAT stages. Results of phase II/III clinical trials are expected soon [3]. Regrettably, so far melarsoprol, which is deadly for 3-10% of the patients that receive it [2], is still the only treatment in severe cases of HAT. Finally, the emergence of resistant strains is an issue that remains to be considered. Taken together, the goal of the WHO to eradicate the disease by 2030 seems to be difficult to achieve.

There is no doubt that a very fine interplay between the host immune system and the parasite must exist during infection. Trypanosomes coexist for months or even years within their host before encephalitic symptoms become visible. Intriguingly, the report of some individuals infected with *T. b. gambiense,* who refused treatment and some years later showed a parasitological clearance [4], indicates that the progression of the disease largely depends on the extent of the host's immune response. In the absence of chemotherapeutic intervention, successful parasite clearance may require a strong pro-inflammatory response, which needs to be counteracted later by anti-inflammatory molecules. Alternatively, something called trypanotolerance may also befall; individuals carrying on the parasite who do not develop signs and symptoms of the disease (latent infections). In these cases, the host's immune response may be sufficiently robust to eliminate most of the parasite load but not strong enough, enabling some parasites to flee and keep infection. Now arises out the question; is it solely the immune response of the host that matters? No indeed. As in every “relationship”, the partner or counterpart also contributes to the outcome of the alliance. That means that features of the parasite also impact the severity of the pathology.

Traditionally speaking, in the case of *T. brucei* the host-parasite interaction comprises the humoral immune response of the host and a parasite's phenomenon called antigenic variation. The former works towards peripheral elimination of the parasite, while the latter enables trypanosomes to change their surface antigen to fool the immune system. However, the fact that the outcome of the disease goes from lethality to “apparently” spontaneous resolution of infection, points out that the complexity of such interaction must include a plenty variety of other molecules acting in favor and against the resolution of the disease. For instance, prostaglandins (PGs), known to be secreted by trypanosomes, may regulate parasite load locally, as some of these molecules induce cell death in the subpopulation of parasites that lost proliferation capability [5]. Interestingly, one of the main PGs, namely PGD_2_, is a potent sleep-promoting molecule produced in the leptomeninges, choroid plexus, and oligodendrocytes in the brain. It is secreted into the CSF as a sleep hormone and causes profound enhancement of sleep [6]. Patients suffering from HAT have increased levels of PGD_2_ in the CSF [7], which correlates with the alteration of the wake/sleep cycle characteristic in HAT. It remains to be clarified whether the PGD_2_ increase in CSF is merely due to the induced inflammatory response by the host or if it also has a parasitic origin since parasites are found in relatively high densities in the meninges during the late stage of the disease.

Despite all advances made over the years about the knowledge of the CNS pathology of HAT, which has been nicely reviewed elsewhere [8–12], processes that are ongoing during the late stage of the disease are at present scarcely understood. Perhaps it occurs as a consequence of the difficulty to access and study affected areas. Most studies were conducted on a cross-sectional basis (i.e., at one specific point in time) and were based on histopathological observations and quantification of the level of some cytokines. Autoptic analyses had shown diffuse microglial hyperplasia, a proliferation of astrocytes, and the formation of microglial nodules [13, 14]. Studies carried on in experimental animal models proved astrocytosis and evidenced neuronal loss in some areas of the brain [15–18]. Another common feature between HAT patients and animal models is the presence of an inflammatory infiltrate in the parenchyma [19, 20]. Although all these observations have provided insights into the neuropathogenesis of HAT, they represent a snapshot of the specimen's status by the time of its preparation. To date, many basic aspects of the late-stage infection need to be clarified. They include but are not limited to: the exact invasion route, the local immune response inside meninges and encephalon, the reaction of neuronal and glial cells against infection, among others. Noteworthy, developments made over the past decade in several new techniques like *in vivo* imaging, single-cell tracking, functional neurophotonics, combined with mouse genetics have become broadly available. They enable the investigation of host-parasite interactions in the brain in real-time, in a longitudinal matter (i.e., follow-up of the same specimen over some time).

Interestingly, in the past years, the use of such *in vivo* techniques has facilitated a more direct investigation of the trypanosome's infection mechanisms in the CNS and the definition of specific features. Several aspects of the meningeal infection have been followed in real-time and evaluated through intravital imaging using a two-photon microscope. In a model for the chronic infection, the entering time of parasites and the infiltration of immune cells in the meninges were video-recorded and described [21]. A significant infiltrate of CD2^+^ T cells, as well as an increase of CD11c^+^ dendritic cells, was determined twelve days post-infection. Besides, video-recordings showing the extravasation of parasites of dural blood vessels as well as interactions between T cells and dendritic cells, suggestive of antigen presentation, were achieved. This study reported parasites located at the dural space. However, in earlier observations parasites were found settled in the pia mater [22]. This aspect would need further investigation since it may have implications in the route used by the parasite to invade the parenchyma.

Of relevance for studies that evaluate the effectiveness of compounds for the late stage of the disease was the development of a non-invasive, *in vivo* imaging method [23]. The method is based on bioluminescence and improves the investigation time needed to identify lead compounds. It enables the determination of the parasite's presence in the whole body in experimental animals. Interestingly, the efficacy of therapeutic interventions other than chemotherapy, for instance, immunomodulation could also be explored using this kind of method.

The use of these cutting-edge tools will empower a better understanding of the pathomechanisms of trypanosome infection on CNS. Of major interest are the events happening below the meninges after and even before parasites reach the CNS. Hopefully, in the near-future important clues about neurological pathogenesis will become clear: a) how and when immune cells in the parenchyma, namely microglia, start the reaction against infection, b) what are the functional properties of astrocytes alongside disease, and to what extent their cross-talk with microglia influence disease progression, c) how neuronal activity pattern is influenced by infection and how it correlates with symptoms, d) which ones are the immunological determinants of the passage of trypanosomes across barriers, among others. This new knowledge will have implications in the development of additional strategies for control and new therapeutics.
